# Impact of systematic capacity building on cataract surgical service development in 25 hospitals

**DOI:** 10.1186/s12886-017-0492-5

**Published:** 2017-06-19

**Authors:** Katherine Judson, Paul Courtright, Thulsiraj Ravilla, Rohit Khanna, Ken Bassett

**Affiliations:** 1 Seva Foundation, 1786 5th St, Berkeley, CA 94710 USA; 2 Kilimanjaro Centre for Community Ophthalmology, Division of Ophthalmology, University of Cape Town, Cape Town, South Africa; 3 Aravind Eye Care System, Madurai, India; 4 LV Prasad Eye Institute, Hyderabad, India; 5 University of British Columbia, Vancouver, Canada

**Keywords:** Capacity building, Ophthalmology, Africa, South Asia, Latin America

## Abstract

**Background:**

This study measured the effectiveness and cost of a capacity building intervention in 25 eye hospitals in South Asia, East Africa and Latin America over 4 years. The intervention involved eye care non-governmental organizations or high-performing eye hospitals acting as “mentors” to underperforming eye hospitals- “mentees” in 10 countries. Intervention activities included systematic planning and support for training and key equipment purchases as well as hospital-specific mentoring which focused on strengthening leadership, increasing the volume and equity of community outreach, improving surgical quality and volume, strengthening organizational and financial management and streamlining operational processes.

**Methods:**

This is a before and after observational study of the impact of this multi-dimensional process on hospital and individual productivity and financial sustainability after 4 years. Mentee hospitals reported data monthly using a standardized template. Key indicators included cataract surgery volume, cataract operations per surgeon, the proportion of direct paying cataract surgical patients, intervention program costs per additional surgery and cost per mentor.

**Results:**

By the end of the study period, the hospitals experienced a 69% average increase (range: −63% to 690%) in cataract surgical volume over baseline with 12 hospitals showing increases over 100%. Twenty-three hospitals experienced a 59% average increase in the number of cataract surgeries per surgeon with 10 hospitals showing increases over 100%. The proportion of paying patients increased in 8 of the 14 hospitals reporting this data. The average mentoring cost per additional surgery for these 25 hospitals was $5.39. An average of $36,489.99 was spent per mentor per year to support their work with mentees.

**Conclusions:**

The intervention resulted in proportionally similar increases in cataract surgical volume and productivity across diverse settings in three distinct geographic regions. Its wide applicability and moderate cost make it an attractive means to rapidly and substantially increase eye care services to meet VISION2020 goals.

## Background

The number of cataract operations in the developing world is far below the level required to take care of new and existing cases [1]. Using the increased volume and high quality approaches initially developed by Aravind Eye Care System (AECS) and LV Prasad Eye Institute (LVPEI) in India, ophthalmologists, along with a well-managed team, can double or triple their cataract surgical volume [2]. Simply demonstrating or describing how to improve productivity by underperforming institutions is seldom enough to stimulate and maintain change [3].

An engaged process with regionally available coaching and training, termed herein ‘capacity building’ has been used by 6 mentoring institutions to accelerate change in eye care services in an effort to meet population need and start on a pathway to organizational and financial sustainability [4].

Capacity building is defined hereinafter as an interactive process whereby an established institution is invited to utilize its staff to engage with and support the internal development of other institutions with which it usually has no financial connection. It is a structured, planned and often altruistic effort to help other institutions wishing to grow and improve productivity and efficiency. Capacity building therefore is distinct from capacity ‘development’ which is an internal process not involving explicit external involvement [5].

The institutions providing capacity building are termed “**mentors**” and hospitals undergoing the capacity building are termed “**mentees**”.^1^ In this study 25 mentee hospitals located in Asia (16), Africa (7), and Latin America (2), were supported by mentor institutions: LV Prasad Eye Institute (LVPEI), Aravind Eye Care System (AECS), Sadguru Netra Chikitalaya **(**SNC), Vivekananda Mission Ashram Netra Niramay Niketan **(**VMANNN) in Asia, Kilimanjaro Centre for Community Ophthalmology (KCCO) in Africa and Visualiza Clinica de Ojos in Latin America. For the purpose of this study all of the mentee hospitals managed by the mentors that had undergone four or more years of the capacity building process by 2014 were included.

The mentees initiated the capacity building process in a variety of ways. Some mentees had long-standing relationships with mentor institutions through their leaders or through ophthalmology training programs; some mentees approached the mentor institution and requested the intervention, while others were recommended by outside governmental and non-governmental organizations.

Several key factors typically influenced a mentor’s decision whether or not to engage with a mentee: [a] leadership capacity or potential [b] willingness/desire to change, [c] willingness by the hospital to establish a separate bank account for the eye unit, [d] some infrastructure/personnel in place. Both private and government hospitals were included.

This study measures the cost and impact of the capacity building intervention on cataract surgical volume, surgical productivity and demand for services.

## Methods

For this research we had three primary questions:

1. Did the capacity building intervention result in an increase in the number of cataract surgical operations per year?

a) by site, compared to baseline averages and provincial or national growth rates?

b) by ophthalmologist compared to baseline?

2) Does the capacity building intervention result in an increase in the total number and proportion of paying cataract surgical patients (‘direct’ walk-in patients) compared to prior years?

3) What are the capacity building costs to increase cataract surgical volume by region?

### Data

Monthly monitoring of surgical activity and program performance at the hospital level begins after the vision building workshop in most settings. Hospitals report their monthly data to the mentor institution. The monthly data sheet collects four main indicators: patient and surgical volume, cataract surgical outcome, hospital financial viability, and human resources utilized.

Mentee reported data were compiled annually. Baseline year was the calendar year prior to active engagement in the capacity building program with the needs assessment visit to the mentee hospital.

National and state cataract surgical rate data were obtained from a variety of sources [6–10]. For almost all countries, data were only available for the four-year time period 2010 to 2013. State-level data provided for India is the 2010–2014 timeframe. Some of the African and Indian interventions began in 2009, however the 2010–2014 comparison data were used. All 25 mentee hospitals were eligible for inclusion of which 23 were included. 2 were not included as CSR (cataract surgical rate) data was not available during the correct time period for mentees in Bangladesh and China (Sichuan Province).

### Key program indicators

1. *Cataract surgical volume*: used as an indicator for overall program growth, reflecting the number of outreach and outpatients examined, their interactions with program staff, and that underwent surgery
2. *Cataract operations per surgeon*: used as an indicator of program efficiency in managing patient flow, operating room procedures, and ancillary staff
3. *Percentage of direct (walk-in), cataract surgical patients who pay for surgery:* used to indicate program acceptance to patients with discretionary income, as well as a proxy for quality and financial sustainability.

Two additional indicators were:

1. T1. the total amount of external funding provided divided by the cumulative amount of additional surgeries performed over the 4 year study
2. The total amount of external funding provided to each mentor divided by the total additional surgeries performed by it’s mentees over the 4 year study.

### Intervention

*The capacity building intervention was not strictly prescribed, nevertheless, all interactions included 4 core components:*

1. *Needs assessment visit*
2. *Vision building workshop resulting in a strategic plan and an action plan*
3. *Ongoing consultation on improvement of services and administration including: training to meet priority needs, support for outreach, and advice on strategic purchases (equipment) and renovations*
4. *Monitoring of key performance indicators (data)*.

### Needs assessment visit

*The capacity building process begins with the mentor institution sending a team (usually made up of at least one ophthalmologist and management staff) for a multi-day visit to the mentee institution designed to understand the current situation and assess the potential for change. This visit includes review of current service delivery and staffing, engagement with community and other stakeholders, organizational and financial systems and observation of clinical practices and outreach. A senior ophthalmologist from the mentor institution spends time with and carefully evaluates the surgical skills of the ophthalmologists in the mentee institutions.*

*The visit usually ends with a review meeting attended by representatives from all levels of clinical and non-clinical staff of the mentee hospital. The goal is to help the staff recognize their growth potential, define limiting issues (bottlenecks) and plan solutions involving cooperation and organizational change.*

### Planning and vision building workshop

*Planning and Vision Building workshops take a variety of formats but share a common goal: facilitating the mentee institution staff to identify what needs to happen, take ownership of the transformation process and to set targets and create a plan of action that will allow the hospital to reach their targets. Activities in the change process include defining new service delivery models, establishing organizational and financial sustainability targets, and introducing new processes into the hospital and outreach systems. Staff from multiple departments of the mentee hospital are often encouraged to attend the vision building workshop so responsibility for the goals is shared among the entire hospital.*

### Ongoing consultation on improvement of services and administration

*Over the next several years, mentors interact with mentees through annual on-site visits and* via *email and telephone. The ongoing dialogue typically deals with service delivery issues and improving organizational and financial sustainability through a structured fee system. Most mentors will on occasion bring mentees together to deal with common issues and review key performance indicators. The ongoing communications and follow-up visits usually involve the same mentor personnel in order to maintain continuity and built relationships.*

*Capacity building involves additional training of various clinical and non-clinical eye care personnel. Initial training often focuses on improving cataract surgical skills, eye unit management and hospital administration and developing outreach program expertise. As the capacity building process matures, in some settings clinical training expands to include subspecialty services and administrative training expands to include auxiliary services such as librarian training and human resources and financial planning.*

*An integral part of the mentoring process is to support some form of regular outreach in order to increase access to and use of eye care services in the catchment area of the mentee institution In some cases (generally not in Africa), outreach activities are supported by a small seed grant until the hospital is able to fund it themselves through new revenue generated through the capacity building process. At times, support for an eye unit manager is required.*

*If necessary, plans for hospital equipment purchase and infrastructure improvements are also developed during the action planning process and executed during the active phase of mentoring. Mentor institutions may provide funding to purchase equipment, and if necessary will work with mentees to secure funding through outside funders.*

*Monitoring of key performance indicators (KPIs)*

*Each mentee is required to submit hospital-level data to their mentor on a monthly basis. These metrics were developed to determine whether or not the change process was having a positive effect on different hospital processes.*

## Results

All 25 mentee hospitals provided data on cataract surgical volume (CSV). The baseline average CSV per year per hospital was 3528 (range: 200–17,897 median: 1432) with substantial variation by geographic region (Table 1). The average CSV increased during years 2 through 4 reaching 69% above baseline by year 4, with the relative increase greatest in Africa (164%) and Latin America (136%) compared with Asia (66%).

**Table 1:** Cataract surgical volume (CSV) per year by geographic region

Geographic region	Baseline CSV	Year 4 CSV	CSV change over baseline (%)
(N hospitals)	N average (range)	N average (range)	
Asia (16)	5294 (248–17,897)	8781 (2010–49,898)	66
Africa (7)	340 (200–5560)	899 (561–1525)	164
Latin America (2)	556 (456–665)	1311 (305–2318)	136
OVERALL	3528 (200–17,897)	5976 (305–49,898)	69

**Fig. 1:**
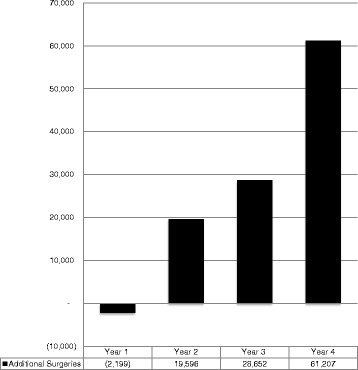
Additional cataract surgeries above baseline over four-year period

The CSV decreased slightly in year 1, increased steadily in years 2 and 3 (Fig. 1) and showed a more marked increase in year 4 due to two high-volume centers in India more than doubling their volume. Asia drove the overall increase in volume accounting for 89,109 (83%) of additional surgeries. Four hospitals did not increase their CSV by year 4, while 8 showed an increase of 200% or more.

In the African countries, the national CSR (defined as number of cataract operations per million people per year) increased slightly or declined in all but one country where a mentee is present (range: −39% - 42%) while the CSV in all but one of the mentee hospitals in this study more than doubled (range: 172%–245% increase) by the end of year 4. The hospital in Burundi saw the greatest overall increase in surgeries of 245% (Table 2).

**Table 2:** National and state cataract surgical rate (CSR) change versus cataract surgical volume (CSV) change at 4 years

Countries	CSR 2010	CSR 2014 [2013]	Change 2010–14/[13] (%)	# of menteeHospitals per country	Baseline cataract surgical volume	Year 4 cataract surgical volume	Change baseline-year 4 (%)
Madagascar	312	373	20	3	1355	3096	128
Ethiopia	468	[480]	[3]	1	236	685	190
Burundi	135	[192]	[42]	1	342	1181	245
Uganda	331	[203]	[−39]	1	250	681	172
Tanzania	543	[562]	[3]	1	200	648	224
Peru	1572	[1130]	[−28]	1	655	2318	254
Honduras	800	[650]	[−19]	1	456	305	−33
Indian States	CSR 2010	CSR 2014	Change 2010–14 (%)	# of menteehospitals per state	Baseline cataract surgical volume	Year 4 cataract surgical volume	Change baseline-year 4 (%)
Andra Pradesh	6782	5805	−14	1	1446	2658	84
Gujarat	12,163	12,920	6	1	5555	7738	39
Haryana	5411	10,467	93	1	5652	5971	6
Odisha	3064	2630	−14	4	32,649	32,078	−2
Madhya Pradesh	5627	6310	12	1	17,897	49,898	179
Uttar Pradesh	3667	3971	8	3	5126	21,772	325
West Bengal	3433	3815	11	1	6025	4816	−20
Assam	1618	2075	28	2	2267	5741	153

The two Latin American countries saw a decline in CSRs (Honduras −19%, Peru −28%) while the CSV in the Peruvian hospital increased by 254% and the Honduran hospital decreased by 33%. The Honduran hospital did not continue to grow after the second year of intervention due a refusal to hire another ophthalmologist.

In India, CSR data is available at the state level. Of the eight states where mentee hospitals are located, CSR decreased in two states by 14%, mildly increased in five (range: 6–28%) and significantly increased in one (93%). Two of the 14 Indian mentees increased their CSV less than the CSR of their state. The other 12 hospitals increased their CSV at more than double the change in CSR (range of increase: 39–325%) (Table 2).

Twenty-three hospitals reported data on the number of operations per surgeon. The baseline average number of ophthalmologists per institution ranged from 1 to 7 depending on region, with 4 times as many surgeries per surgeon in Asia (average: 1704) compared with Latin America (average: 370) and Africa (average: 340). All regions increased the number of surgeries per surgeon with the greatest relative increase in Africa (131% increase) (Table 3). By year four, 4 hospitals (3 India, 1 Latin America) did not increase the number of surgeries per surgeon. Of the 19 hospitals showing an increase, more than half increased 100% or more (5 Africa, 1 Latin America, 4 Asia).

**Table 3:** Cataract surgeries per ophthalmologist per year over 4 years, by geographic region

Geographic region	Baseline Surgeons	Baseline cataract surgeries per surgeon	Year 4 surgeons	Year 4 cataract surgeries per surgeon	Change in surgeries per surgeon (%)
	N average (range)	N average (range)	N average (range)	N average (range)	
Asia (14)	3 (1–7)	1704 (248–8222)	3 (1–7)	2602 (402–7128)	53
Africa (7)	1 (1–1)	340 (200–566)	1 (1–1)	786 (561–1181)	131
Latin America (2)	1 (1–2)	370 (327–456)	2 (1–2)	580 (305–772)	56
OVERALL	2 (1–7)	1444 (200–8222)	2.5 (1–7)	2217 (305–7128)	54

Fourteen mentees (13 from Asia, 1 from Latin America) reported the number of patients who come directly to the hospital and paid all or a portion of the cataract surgical fees. Countries in Africa did not provide data on walk-in paying patients. The number of direct paying patients showed an average increase of 61% by year 4 (Table 4). Eight of the 14 hospitals increased the percent of paying patients (range: 12%–1397%) while 6 decreased (12%–64%) by the end of the fourth year of intervention. Of the 8 hospitals that increased the percent of paying patients by year four, 4 hospitals increased by 200% or more. The hospital from Latin America saw a decrease in number of paying patients.

**Table 4:** Proportion of paying cataract patients over 4 years by geographic region

Geographic region (N hospitals)	Baseline paying cataract patients N average (range)	Proportion of cataract patients (%)	Year 4 paying cataract patients N average (range)	Year 4 proportion of cataract patients (%)	Change in proportion of cataract patients (%)
Asia (13)	675 (112–1457)	12	1870 (308–3857)	21	66
Latin America (1)	352 (n/a)	54	546 (n/a)	24	−56
OVERALL (14)	652 (112–1457)	13	1775 (308–3857)	21	61

Seva or the Swiss Lions paid an average of $5.39 (range: 55¢ - $13.89) per additional surgery (Table 5). The highest “return on investment” by mentor was 55¢ per additional surgery across their 2 mentees. By geographic region, Asia saw the highest return on investment with $4.16 per additional surgery while Latin America saw the lowest with $13.89 per additional surgery.

**Table 5:** Funding per cumulative additional cataract surgeries over 4 years by geographic region

Geographic region (N mentors)	Amount spent in USD	Cumulative additional surgeries over 4 years	Amount spent per additional surgery in USD
Asia (4)	$ 570,492.99	137,085	$ 4.16
Africa (1)	$ 229,562.39	20,060	$ 11.44
Latin America (1)	$ 75,704.43	5451	$ 13.89
OVERALL	$ 875,759.81	162,596	$ 5.39

## Discussion

The capacity building intervention focused on strengthening organizational management, leadership, team building, equitable access and use of services, financial cost-recovery, and quality of care. How this transpired depended upon the local context; while general principles were followed in all settings considerable variation in setting of priority interventions was noted. In most of the South Asian settings the focus was on providing high quality surgery to all sectors of the population, introducing a tiered patient paying system and reorganizing patient flow for maximum efficiency. In African settings priority interventions focused on strengthening basic organizational, personnel, and financial management systems and designing practical outreach programs. Because the services were provided equitably and sustainably through service fees, they met most of the criteria of effective [11] and systematic [12] capacity building.

The capacity building intervention itself varied substantially from mentee to mentee and mentor to mentor, depending upon the local context and needs. Some mentee facilities only needed minimal interventions such as re-orientation and team building strategies, others required physical renovations and equipment, while most required outreach programs and clinical and management training. Nevertheless, in all settings, raising production targets and changing institutional attitudes to growth was a big part of the capacity building program. However, target setting for growth was more of an internal exercise to increase supplies, training, and efficiency, not an explicit attempt to meet population need. In all of the settings the need for large increases in eye care services, including cataract surgery, was simply accepted as a background reality in the planning process.

Hospitals and eye units worldwide use cataract surgical volume (CSV) as an indicator of overall eye care activity level. It reflects hospital performance and efficiency including patient choice and acceptance of surgery as well as the quality of ophthalmic professionals, patient experience, equipment and supplies. CSV directly depends on the number of patients attending hospital outpatient departments and community outreach activities, often termed diagnostic/screening ‘camps’. Attendance at outpatient and outreach, in turn, reflects interaction with the service population regarding eye diseases and their awareness of treatment options. While mentee hospitals and eye units gathered and used these statistics for their own improvement, the complexity and variability was too great to include in this initial, broad level, assessment study.

The capacity building intervention was systematic [12] it resulted in an initial decrease in cataract surgical volume in 11 of 25 institutions due to key clinical staff undergoing training off site, physical alterations to the hospital building and installation and training on new equipment, as well as service populations learning to accept paying fees for previously free services (though free services were still provided for patients too poor to pay). Despite widely diverse population density, disease prevalence, and eye care infrastructure [13, 14] the capacity building intervention show a substantial increase in cataract surgical volume by year 4 in Africa (164%) Asia (66%) and Latin America (136%).

The proportional increase in CSV observed exceeded the change in cataract surgical rate (CSR) over the same time period in all but three jurisdictions [6–10]. CSR is a widely used, population-based measure, while CSV is simply the number of operations per year in an institution, without the population denominator. Nevertheless, the comparison provides a reasonable way to assess mentee growth rate to background eye care system growth in the same geographical area, thereby controlling for broad political and economic changes during the study period. CSR remains unchanged or declines in settings such as Madagascar and Tanzania where most eye care services are part of government hospitals where eye care is a low priority, equipment is not affordable, and active outreach does not occur. In settings such as India, CSR is steadily rising, albeit much slower than the mentee institutions studied here, because of substantial government and private investment in establishing high quality eye care services and broad distribution of eye care services to the primary health care level.

Surgical productivity (defined herein as cataract operations per ophthalmologist or cataract surgeon) also increased in all regions by year 4. This reflects improvement in a range of internal hospital features including training, clinical protocols, patient flow within the hospital and operating room, and reallocating less-skilled tasks from the ophthalmologist to the rest of the eye care team [15]. Surgical productivity is also dependent upon the burden of disease in the population and there is strong evidence that the incidence of cataract (age and sex adjusted) in many African populations is two to four times lower than in other populations [14].

Direct, walk-in patients who pay for some or all of their care reflect a number of different conditions, some hospital related and some related to the local context. Where transport systems are efficient and effective “direct-paying patients” or their family members have chosen to seek care at the mentee facility over other local and distant treatment options and to use their own money to pay for services. This choice indicates good hospital reputation regarding quality, cost and efficiency of services. Eye care institutions such as AECS in India achieve a cash surplus with only 30–40% of their cataract patients as direct paying patients [16]. As has been shown in different settings in Africa, direct paying patients are generally fewer in number as compared to South Asia. Poor transport systems, long distances, advanced age at development of cataract, and inadequate social support all contribute to a relatively low proportion of direct paying patients [17–19].

Of the 14 hospitals that reported direct paying patient information, more than half reported that paying patients made up 15% or less of total cataract surgical patients prior to the capacity building intervention. Many of these hospitals were founded as charity hospitals that provided services to the poor and therefore did not seek payment from patients leaving the hospitals vulnerable to changes in external funding. In the African countries, government hospitals (4 of the 7) had minimal funding for eye care, mostly consisting of covering salaries. In most instances, early discussions between mentors and mentees included conversations regarding implementation of fee structures and payment options suitable to the hospital context and fiscal demographics of their catchment area. Restructuring patient fees to provide a tiered payment system (particularly in South Asia and Latin America) includes taking into account what top, middle and low income earners would be able to pay for surgical intervention based on the most up-to-date demographic information available for the hospital catchment area and building pricing structures on that while still providing an option for free surgery for those who cannot afford the least expensive option.

The assessment of the return on investment of this capacity building process would suggest that the South Asian sites had the best value-for-money. As noted by Lewallen and Thulsiraj [13] there are a considerable number of factors that limit the ability of directly apply practices from India to different settings in Africa. Comparisons within similar settings may be a more valuable approach to assessing value-for-money. Nevertheless, in all of these settings capacity building proved to be a relatively inexpensive form of increasing the number of people who received cataract surgery.

### Limitations

The capacity building intervention was associated with increased cataract surgical volume and productivity in diverse settings. However, prospective controlled studies are needed to prove the impact on individual institutions in each region. State and national CSR data were used for historical comparison. Although crude indicators, they provide the best available way to capture broad trends in national and state eye health system development

A much better assessment of mentee impact would be captured by measuring cataract surgical coverage (operated cataract as a proportion of operable plus operated cataract) and the more recent ‘effective’ cataract surgical coverage (operated cataract and a good outcome as a proportion of operable cataract plus operated cataract) as applied in Ramke, et al. [20]. While not possible herein with these early mentees, this approach is becoming more and more feasible with the increasing number of Rapid Assessment of Avoidable Blindness survey studies in all of these settings.

Data on the quality of cataract surgery, although gathered by all the mentee hospitals, were not reported in this study. In this program evaluation, high quality surgery was considered a necessary variable in higher level indicators such as the number of services provided and the number and proportion of patients willing to pay for services.

## Conclusions

In summary, the capacity building interventions resulted in a proportionally similar increase in cataract surgical volume and productivity across diverse settings in three distinct geographic regions. Its wide applicability, and moderate cost make it an attractive means to rapidly and substantially increase eye care services to meet VISION 2020 goals.

## Annex 1: List of Mentor and Location of Mentee Institutions Involved in this Study

**Kilimanjaro Center for Community Ophthalmology (KCCO), Tanzania**

Madagascar [3]

Ethiopia [1]

Burundi [1]

Tanzania [1]

Uganda [1]

**Lions Aravind Institute for Community Ophthalmology (LAICO), India**

India [5]

China [1]

**LV Prasad Eye Institute (LVPEI), India**

India [4]

**Sadguru Netra Chikitalaya (SNC), India**

India [2]

**Visualiza Eye Care System, Guatemala**

Peru [1]

Honduras [1]

**Vivekananda Mission Ashram Netra Niramay Niketan (VMANNN), India**

India [3]

Bangladesh [1]

## Abbreviations

AECS: Aravind Eye Care System
CSR: Cataract surgical rate
CSV: Cataract surgical volume
KCCO: Kilimanjaro Centre for Community Ophthalmology
KPI: Key performance indicator
LVPEI: LV Prasad Eye Institute
SNC: Sadguru Netra Chikitalaya
VMANNN: Vivekananda Mission Ashram Netra Niramay Niketan

## Footnotes

1 Please see annex 1 for a complete list of “mentor” and locations of “mentee” institutions involved in this study
